# Is It Enough to Be an Extrovert to Be Liked? Emotional Competence Moderates the Relationship Between Extraversion and Peer-Rated Likeability

**DOI:** 10.3389/fpsyg.2018.00804

**Published:** 2018-05-23

**Authors:** Dorota Szczygiel, Moïra Mikolajczak

**Affiliations:** ^1^Sopot Faculty of Psychology, SWPS University of Social Sciences and Humanities, Sopot, Poland; ^2^Department of Psychology, Université catholique de Louvain, Louvain-la-Neuve, Belgium

**Keywords:** personality, trait emotional intelligence, emotional competence, adolescents, peer relations, social status, peer acceptance

## Abstract

Likeability represents one of the aspects of social status in a peer group and refers to the extent to which one is accepted, preferred by others, and perceived as a likeable companion. Previous research has demonstrated that likeability is partly determined by dispositional factors. One body of research shows that variance in likeability across individuals can be traced to personality traits, mainly extraversion and agreeableness. Another expanding body of research demonstrates that success in achieving peer acceptance is determined, in some part, by the emotional competencies (ECs) of an individual. In an attempt to combine these two approaches and to clarify some inconsistencies in the results concerning the personality–likeability relationships, this study was designed to examine the interactive effect of adolescents’ personality traits (i.e., extraversion and agreeableness) and ECs on peer-rated likeability in adolescence. A sample of 230 adolescents (47% female) from two comprehensive secondary schools in Poland completed measures of personality traits and ECs, as well as a sociometric assessment of likeability in their classrooms. The results demonstrated that interpersonal EC acts as a moderator in the relationship between extraversion and peer-rated likeability. Specifically, extraversion predicted greater likeability among adolescents with high interpersonal EC but not among adolescents with low interpersonal EC. The study yielded new insights into the determinants of likeability, as it demonstrates that adolescents need to be both extrovert and possess high interpersonal EC in order to be judged highly likeable by their peers. It also bears practical implications for the improvement of adolescents’ position and acceptance within their peer group. The results suggest that encouraging “rejected” adolescents to reach out to others in an extrovert fashion is necessary but insufficient to increase their likeability. Improving their interpersonal EC is also necessary. The observation that higher levels of interpersonal EC helps adolescents to achieve higher acceptance in their peer group suggests the need to implement school training programs aimed at improving the core ECs (identification, understanding, expression, regulation and use of emotions).

## Introduction

Much of human behavior is directed toward creating and maintaining social relationships with other people (Baumeister and Leary, 1995). The importance of belonging to a meaningful group is especially pronounced during adolescence (Steinberg, 2002; LaFontana and Cillessen, 2010). As adolescents begin to demand more autonomy, the influence of parents decreases at the expense of peer influence. The peer group becomes the psychosocial context within which adolescents experience social acceptance and rejection for the first time (Bukowski et al., 1993). For many adolescents, achieving high status in their peer group may be more important than achieving academic goals (Eccles and Roeser, 2011).

Indeed, attaining high status in and being accepted by the peer group serves important functions: the social status of adolescents increases their well-being (Newcomb et al., 1993), social (Meuwese et al., 2017) and emotional (Brendgen et al., 2005) functioning, as well as successful adaptation into school (Wentzel, 2003). It also increases access to potential mates and social support amidst adversity (Cillessen and Rose, 2005). Finally, it predicts future adjustment (Sandstrom and Cillessen, 2006) and better social functioning during adult life (Ostberg, 2003).

The social status of adolescents encompasses two categories: likeability and popularity (Cillessen and Rose, 2005). Likeability refers to the extent to which one is accepted, preferred by others and viewed as a likeable mate, whereas popularity refers to visibility, prestige and being central in the peer group (Cillessen and Rose, 2005). Likeable individuals are considered as friendly, prosocial, cooperative and exhibiting low levels of aggressive behaviors, whereas popular individuals are considered as friendly and attractive, but also dominant and occasionally aggressive (Engels et al., 2017; Lansu and Troop-Gordon, 2017). The correlations between likeability and popularity are modest (e.g., van der Linden et al., 2010), meaning that being popular does not necessarily translate into being liked, and being liked does not necessarily translate into being popular (Sandstrom and Cillessen, 2006; Boor-Klip et al., 2017).

This study focused on likeability and the effects of an individual’s dispositions on peer-rated likeability in adolescence. The following sections review previous research focusing on the two relationships: personality and likeability, and emotional competencies (ECs) and likeability. In an attempt to bridge these two lines of research, this study was designed to examine the interactive effect of adolescents’ personality traits (i.e., extraversion and agreeableness) and ECs on peer-rated likeability.

## The Big Five Personality Traits and Likeability

In the Big Five model of personality (McCrae and John, 1992), extraversion and agreeableness are considered to capture the social aspects of personality (Jensen-Campbell et al., 2002; Ozer and Benet-Martinez, 2006). *Extraversion* refers to the extent to which people are assertive, active, cheerful, talkative and energetic (Costa and McCrae, 1992). Individuals high on extraversion are predisposed to experience positive emotions (Watson and Clark, 1997) and have a preference for seeking, engaging in and enjoying social interactions, whereas individuals low in extraversion prefer to spend more time alone and tend to be reserved, withdrawn and quiet in social settings (Costa and McCrae, 1992). *Agreeableness* refers to the extent to which people are motivated to achieve interpersonal intimacy (Costa and McCrae, 1992). People who score high on agreeableness are characterized by being caring, altruistic, tender-minded (Costa and McCrae, 1992) and responsive to the needs of others (Tov et al., 2016), whereas people who score low on agreeableness are characterized by being manipulative, self-centered and occasionally ruthless (Digman, 1990). Furthermore, highly agreeable individuals, compared to their less agreeable counterparts, tend to engage in more prosocial behaviors, respond more constructively to interpersonal conflicts, cooperate more during group tasks, and expend more effort to suppressing negative emotions in social situations (Tobin et al., 2000; Jensen-Campbell and Graziano, 2001).

There is evidence that extraversion and agreeableness play an important role in predicting peer acceptance in adolescence. Jensen-Campbell et al. (2002) reported positive correlations between both personality factors and peer acceptance in early adolescence. These results were corroborated by Lubbers et al. (2006), who demonstrated a positive relationship between extraversion and peer acceptance for boys and girls, and a positive relationship between agreeableness and peer acceptance for girls, but not for boys. Wolters et al. (2014) observed that peer acceptance correlated significantly more strongly with extraversion than with agreeableness. Likewise, van der Linden et al. (2010) reported positive correlations between both extraversion and agreeableness, and classroom ratings of likeability, but agreeableness lost its significance in predicting peer-rated likeability when other personality Big Five dimensions were examined simultaneously. Among college students, extraversion, but not agreeableness, correlated positively with peer reports on the quality of interpersonal relationships, that is, received social support (Lopes et al., 2004). In another study, Lopes et al. (2005) reported a significant and positive correlation between agreeableness and peer nominations of liking, but the correlation between extraversion and peer nominations was insignificant.

Overall, despite some inconsistencies in the results of the above-mentioned studies, it appears that the more extroverted and agreeable adolescents are, the more they are accepted by their peers. The inconsistency of the results, however, suggests that the relationship between personality and peer-rated likeability is more complex, and that the presence of moderators in this relationship may need to be taken into consideration. This study will look more closely at this issue by analyzing whether combinations of personality traits with other individual dispositions have unique effects on the prediction of likeability. We believe that individual differences in ECs constitute a particularly fruitful direction: an expanding body of research (presented in the following section) demonstrates that success in achieving peer acceptance is, in part, determined by the ECs of an individual.

## Emotional Competence and Likeability

Emotional competence (EC) – also labeled as “emotional intelligence” (EI) or “emotional skills” (ES) – refers to the extent to which people functionally identify, express, understand, regulate and use their own and others’ emotions (Saarni, 1990; Mayer and Salovey, 1997; Petrides and Furnham, 2003; Brasseur et al., 2013; Petrides et al., 2016). The term EI is more common to designate these individual differences, but the term EC seems more consistent with recent results showing that, unlike intelligence, these competences can be lastingly improved via relatively short trainings (Kotsou et al., 2011; Nelis et al., 2011). For this reason, and because in this study we operationalised EC through the Profile of Emotional Competence (PEC), the term “emotional competence” will be used hereafter. The reason why we used the PEC is twofold: first and foremost, it is the only EC measure that distinguishes clearly between the intrapersonal and interpersonal facets of each dimension. Second, it does not contain subscales that would correlate too much with extraversion and agreeableness, thereby decreasing the risk of collinearity.

Previous research has shown that the level of EC is associated with self-reported and peer-rated sociability in both children and adolescents. English and Dutch pupils who score high on EC receive more nominations from their classmates for being kind and cooperative, and for having leadership qualities (e.g., Mavroveli et al., 2007, 2009; Mavroveli and Sánchez-Ruiz, 2011). These results were replicated in a prospective design with adolescents (Frederickson et al., 2012). In addition to facilitating prosocial behavior, there is also evidence that EC decreases antisocial behavior: pupils who score high on EC present less externalizing behaviors (aggression and delinquency; Santesso et al., 2006) and receive less nominations for being bullies (Mavroveli and Sánchez-Ruiz, 2011). There is also growing evidence showing that EC is causally involved in these outcomes: when EC is enhanced through training, empathy increases and antisocial behaviors decrease (e.g., Castillo et al., 2013; Pour et al., 2014). In Castillo et al. (2013), adolescents in the EC training group (*N* = 361) reported lower levels of physical/verbal aggression, anger and hostility compared to students in the control group (*N* = 229). Additionally, the EC program was particularly effective in increasing males’ empathic abilities. Pour et al. (2014) found the same results in a smaller sample (*N* = 40) of primary school students.

## The Current Study

To our knowledge, no study has investigated how personality traits and intrapersonal and interpersonal ECs might work together in predicting adolescents’ likeability in their peer group. We hypothesized that personality traits and ECs would both be primary predictors of peer-rated likeability in adolescence. We further hypothesized, however, that the links between personality traits and peer-rated likeability would be moderated by ECs. Therefore, we propose that the association of extraversion and agreeableness with peer acceptance depends on the levels of ECs. Specifically, we propose that the relationships between extraversion and agreeableness and likeability will be strengthened among those with higher ECs than among those with lower ECs.

The aim of the study was threefold: (1) to replicate the previously demonstrated relationships between personality and EC on peer-rated likeability (e.g., Jensen-Campbell et al., 2002; Lubbers et al., 2006; van der Linden et al., 2010; Mavroveli et al., 2007, 2009; Mavroveli and Sánchez-Ruiz, 2011; Frederickson et al., 2012); (2) to extend the results regarding EC by analyzing the respective contribution of intra- and inter-personal EC to this effect; and, most importantly, (3) to examine the interactive effects of personality (extraversion and agreeableness respectively) and EC on likeability.

We propose the following hypotheses: extraversion and agreeableness are both positively related to peer-rated likeability (H1); intra- and inter-personal ECs are both positively related to peer-rated likeability (H2); given the divergent results found in Belgium and Japan on the respective contribution of intra- and inter-personal ECs to social relationships, and because Poland lies in between these two countries in terms of social-related cultural values (Hofstede et al., 2010; Brycz et al., 2015), we had no *a priori* hypothesis on the respective contribution of intra- and inter-personal ECs to likeability in Poland. ECs moderate the relationship between extraversion and peer-rated likeability in such a way that the relationship is stronger among those with higher ECs than among those with lower ECs (H3); ECs moderate the relationship between agreeableness and peer-rated likeability in such a way that the relationship is stronger among those with higher ECs than among those with lower ECs (H4).

## Materials and Methods

### Participants

The participants consisted of 230 students (47% female) from nine first- and second-grade classrooms of two upper secondary schools in Poland (the north-western part of Poland; Pomeranian District). The mean age was 15.97, *SD* = 0.67, ranging between 15 and 17. The average classroom size in this study comprised 26 students, ranging between 20 and 33. The education system in Poland consists of 6 years of primary school, followed by 3 years of lower secondary school, and then three or 4 years (depending on the type of school) of upper secondary school. Although only primary and lower secondary school are compulsory, the vast majority of students continue their studies in upper secondary school. The ethnic composition of the sample was solely Polish.

### Measures

#### Likeability

Likeability was assessed using the widely used peer nomination method (van der Linden et al., 2010; Garcia Bacete and Cillessen, 2017). Each participant could nominate three classmates for each of four questions: (1) most liked; (2) most supportive; (3) most cooperative; and (4) most admired. The average peer-rated likeability score was computed by summing up the peer nominations across all questions and dividing them by four (i.e., the total number of questions). Subsequently, the average likeability score was standardized to *z*-scores within classrooms, which is a commonly used method to control for differences in classroom size (e.g., Ciarrochi and Heaven, 2009). In addition, in order to make sure that the peer nominations form one coherent factor, a principal components analysis with oblimin rotation was performed. The results revealed that all four questions loaded on one factor with an eigenvalue of 3.252, accounting for 81.30 per cent of the variance. All factor loadings exceeded 0.87.

#### Personality Traits

Personality traits were assessed using Costa and McCrae’s (1992) Personality Inventory NEO-FFI (Polish adaptation by Zawadzki et al., 1998). The NEO-FFI comprises 60 self-descriptive statements, 12 for each of the five dimensions of personality: neuroticism (e.g., “I am not a worrier”; reversed), the tendency to experience negative emotions, such as anxiety and depression and cope poorly in response to stressors; extraversion (e.g., “I like to have a lot of people around me”), the tendency to experience positive emotions, to be sociable, active, cheerful and in search of stimulation; openness to experience (e.g., “I often try new and foreign foods”) reflects individuals who are open, imaginative, creative and willing to explore new ideas; agreeableness (e.g., “I try to be courteous to everyone I meet”), the dimension of interpersonal relations, characterized by altruism, modesty, trust and cooperative tendencies; and conscientiousness (e.g., “I keep my belongings clean and neat”), the tendency to be organized, persistent, reliable, and a follower of rules and ethical principles. Items are rated on a five-point Likert scale, ranging from 1 (*completely disagree*) to 5 (*completely agree*). Scale scores were formed by averaging the responses to the items associated with each personality dimension, after appropriate items were reversed. As scores on each scale increase, individuals are describing themselves as scoring higher on each personality dimension.

#### Emotional Competence

Intra- and inter-personal emotional competences were assessed with the PEC (Brasseur et al., 2013). This instrument consists of 50 five-point items (25 items for each dimension) with answers on a five-point Likert scale, ranging from 1 (*completely disagree*) to 5 (*completely agree*) and provides an intra-personal EC score, an inter-personal EC score and a total EC score. Examples of items are: “during an argument, I can’t identify if I am sad or angry (reversed)” and “I am usually able to influence the way other people feel”, for intra- and inter-personal EC, respectively. The validation process of the PEC (see Brasseur et al., 2013) has shown the satisfactory psychometric properties of the questionnaire: both subscales showed high internal consistency, the two-factor structure was confirmed, and concurrent and predictive validity were as expected, on subjective and objective criteria alike (see also Mikolajczak et al., 2014). In the current analyses, we specifically focused on the intra- and inter-personal EC scores. Scores for intra- and inter-personal ECs were calculated by averaging the responses to the items associated with each EC dimension, after appropriate items were reversed.

#### Procedure

Trained research assistants (i.e., psychology students who volunteered to take part in this project) administered all measures in each of the nine classrooms. The participants were given a brief introduction to the project and were assured that the collected data would be kept confidential and only used for research purposes. The questionnaires were administered in paper-and-pencil format with written instructions. Participants first filled out the NEO-FFI and then the PEC. Once the questionnaires were completed, they were returned to the research assistants. Subsequently, the participants were asked to nominate three classmates for each of four questions. When sociometric ratings were completed, protocols were collected by the research assistants. The first part of the procedure (i.e., questionnaires) required the participants to provide their names and surnames, while the second part (i.e., sociometric nominations) was anonymous. The average time participants spent on completing all measures was about 35 min. No compensation was awarded to the participants. Due to the excellent collaboration of the schools and teachers, and low absenteeism in classes, the participation rate was 100% in all classrooms except three, in which the participation rates were 85, 86, and 94%. For students who were absent, the study was conducted on a later date (within 3 weeks after the data in the classrooms were collected). Finally, out of 236 possible participants, data were collected from 230 students (i.e., the total participation rate was 96%). The study was reviewed and approved by school heads and teachers. Parental consent was obtained prior to data collection, during parent-teacher conferences at school. Students participated on a voluntary basis; no one refused to participate. All study procedures were approved by the Ethics Committee of the SWPS University of Social Sciences and Humanities, Faculty in Sopot (Ref. No. WKE-S-28-I-36), by which human subjects’ protection is ensured.

## Results

### Preliminary Results

**Table 1** contains the means, standard deviations, internal consistency coefficients (Cronbach’s α) and intercorrelations of all the variables measured. The pattern of bivariate correlations between the variables was in line with our expectations and fully supports our H1 and H2. First, extraversion and agreeableness were both significantly and positively associated with peer-rated likeability. Second, intra- and inter-personal ECs were both significantly and positively associated with likeability. Note that while the bivariate correlation between extraversion and likeability was significantly stronger than the correlation between agreeableness and likability (*z* = 2.05, *p* < 0.05), the bivariate correlation between inter-personal ECs and likeability was not statistically stronger than the correlation between intra-personal ECs and likeability (*z* = -0.35, ns). Both intra- and interpersonal ECs were positively associated with extraversion, agreeableness, openness to experience and conscientiousness, and negatively associated with neuroticism. We also observed a small, albeit significant, negative correlation between neuroticism and likeability, and positive correlations between age and both intra- and inter-personal ECs scores.

**Table 1:** Internal consistency reliability (Cronbach’s α), means, standard deviations and intercorrelations among all study variables.

Variables	M	SD	1	2	3	4	5	6	7	8	Gender	Age
(1) Likeability	0	0.98	(0.92)	0.34^∗∗∗^	0.16^∗^	–0.17^∗∗^	0.03	0.10	0.30^∗∗∗^	0.33^∗^	–0.05	0.09
(2) Extraversion	2.54	0.56		(0.79)	0.23^∗∗^	–0.24^∗∗∗^	0.10	0.16^∗^	0.40^∗∗∗^	0.42^∗∗∗^	–0.04	0.10
(3) Agreeableness	2.41	0.51			(0.79)	–0.19^∗∗^	0.27^∗∗∗^	0.21^∗∗^	0.29^∗∗∗^	0.35^∗∗∗^	–0.05	0.12
(4) Neuroticism	1.67	0.69				(0.85)	–0.10	–0.26^∗∗∗^	–0.40^∗∗∗^	–0.30^∗∗∗^	–0.13^∗^	–0.05
(5) Openness to experience	2.38	0.44					(0.80)	0.12	0.31^∗∗∗^	0.25^∗∗∗^	–0.11	0.04
(6) Conscientiousness	2.65	0.57						(0.82)	0.30^∗∗∗^	0.24^∗∗∗^	–0.05	–0.03
(7) Intrapersonal ECs	3.25	0.46							(0.86)	0.56^∗∗∗^	–0.14^∗^	0.15^∗^
(8) Interpersonal ECs	3.43	0.53								(0.91)	–0.15^∗^	0.16^∗^

*Diagonal values are the internal consistency estimates for each scale. Gender is coded 0 = female, 1 = male.*

*^∗^*p* < 0.05, ^∗∗^*p* < 0.01, ^∗∗∗^*p* < 0.001 (all two-tailed significance tests).*

### Main Results

In order to examine the main and interactive effects of personality traits and ECs on peer-rated likeability, a moderated hierarchical regression analysis was conducted. The variables were entered into the regression model in four steps. In order to rule out the possibility that associations between personality traits, ECs and likeability could be confounded by socio-demographic characteristics, the participants’ gender (dummy coded: female = 0, male = 1) and age were entered in the first step as control variables. Extraversion and agreeableness were entered in the second step. Intra- and inter-personal ECs were entered in the third step. In the fourth step, in order to examine whether the main effects of extraversion and agreeableness were moderated by ECs, four interaction terms were entered, which were products of personality traits and ECs. These terms were: extraversion × intra-personal EC; extraversion × inter-personal EC; agreeableness × intra-personal EC; and, agreeableness × inter-personal EC. Personality traits and ECs were centered prior to creating interaction terms, rendering the beta-weight of the interaction terms more directly interpretable (Cohen et al., 2003). The data were examined for multicollinearity between independent variables using the tolerance and the variance inflation factor (VIF). Analyses indicated that there was no concern for multicollinearity, as all VIFs were below 2.5 (e.g., O’Brien, 2007). All statistical analyses were performed using the SPSS version 24 statistical package.

The results are depicted in **Table 2**. As can be seen, the full model explains 18% of the variance in peer-rated likeability. The results showed that none of the variables entered in Step 1 emerged as a significant predictor of likeability. In Step 2, when extraversion and agreeableness were entered, the amount of variance explained increased significantly (Δ*R^2^* = 0.11, *p* < 0.001), but only extraversion emerged as a significant predictor of likeability. When ECs were entered in Step 3, the amount of variance explained increased significantly (Δ*R^2^* = 0.04, *p* < 0.01), but only inter-personal ECs emerged as a significant predictor of likeability. Extraversion remained significant. These results indicate that inter-personal ECs predicted likeability above and beyond personality traits. In Step 4, when interaction terms were entered, the amount of variance explained increased significantly (Δ*R^2^* = 0.03, *p* < 0.05), but only the interaction of extraversion and inter-personal ECs emerged as a significant predictor of likeability. Extraversion and inter-personal ECs remained significant.

**Table 2:** Results of the moderated hierarchical regression analyses on the influence of extraversion, agreeableness and emotional competence on peer-rated likeability.

Variables	Step 1 β	Step 2 β	Step 3 β	Step 4 β
Gender	–0.05	–0.03	0.01	–0.01
Age	0.09	0.05	0.02	0.02
Extraversion		0.32^∗∗∗^	0.22^∗∗^	0.23^∗∗^
Agreeableness		0.08	0.01	0.05
Intrapersonal ECs (Intra EC)			0.10	0.07
Interpersonal ECs (Inter EC)			0.18^∗^	0.22^∗∗^
Extraversion × Intra EC				–0.09
Extraversion × Inter EC				0.29^∗∗^
Agreeableness × Intra EC				0.10
Agreeableness × Inter EC				–0.11
*R^2^* (adjusted)	0.00	0.11	0.15	0.18
Δ*R^2^*	0.00	0.11	0.04	0.03

*^∗^*p* < 0.05 ^∗∗^*p* < 0.01 ^∗∗∗^*p* < 0.001.*

**FIGURE 1:**
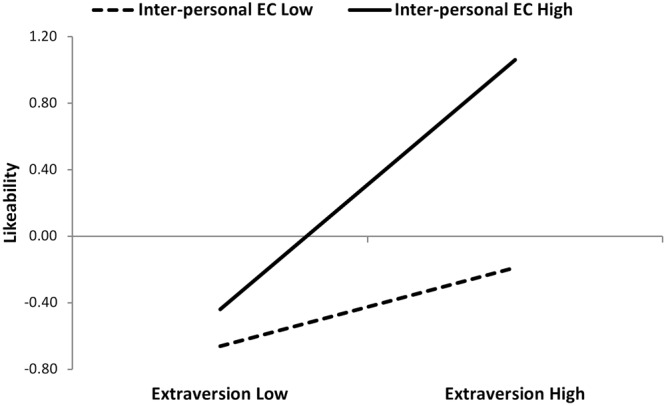
Peer-rated likeability as a function of extraversion and interpersonal emotional competence. Low extraversion is defined as a mean -1 standard deviation from the mean; high extraversion is defined as a mean +1 standard deviation. Note that this high/low split is for illustrative purposes only; the moderation analyses conducted use all variables as continuous variables.

In order to examine further the shape of the interaction, the simple slope procedure recommended by Aiken and West (1991) was employed. Therefore, the relationship between extraversion and interpersonal ECs was plotted, comparing students who scored more than one standard deviation above and below the average level of interpersonal ECs. As shown in **Figure 1**, extraversion predicted greater likeability among students high in interpersonal ECs (β = 0.60, *p* < 0.01) but not among students low in interpersonal ECs (β = -0.06, *p* = 0.78). In other words, extraversion only increases likeability for students high in interpersonal ECs. These results support H3. Note that the opposite is also true: interpersonal ECs only increase likeability among students high in extraversion. H4, predicting the moderating effect of ECs in the relationship between agreeableness and peer-rated likeability, was not confirmed.

## Discussion

The present study aimed to investigate the interactive effects of personality (extraversion and agreeableness) and emotional competence (intra- and interpersonal ECs) on peer-rated likeability. The results showed that both extraversion and agreeableness were significantly and positively related to peer-rated likeability, which is consistent with previous studies (e.g., Jensen-Campbell et al., 2002; Lubbers et al., 2006). It should be noted, however, that extraversion and agreeableness were not equally predictive of likeability: extraversion was more strongly associated with this variable than agreeableness. First, the correlational findings show that the extraversion–likeability relationship was significantly stronger than the agreeableness–likeability relationship. Similar results were reported by Wolters et al. (2014). Second, when extraversion and agreeableness were entered simultaneously into the regression model, agreeableness lost its significance in predicting likeability. Similar results were demonstrated by van der Linden et al. (2010).

We also replicated and extended findings from previous research on the relationship between EC and likeability. We demonstrated that both intra- and interpersonal ECs positively correlated with likeability, which is consistent with previous studies (Petrides et al., 2006; Mavroveli et al., 2007, 2009). Interpersonal EC, however, seems more important, as intrapersonal EC lost its significance in predicting likeability when both intra- and interpersonal ECs were entered simultaneously into the regression model. Moreover, both intra- and interpersonal ECs were associated with personality traits, that is, ECs were positively related to extraversion, agreeableness, openness to experience and conscientiousness, and negatively related to neuroticism, which is consistent with previous research (e.g., Nozaki and Koyasu, 2016). In addition, interpersonal EC predicted likeability above and beyond extraversion and agreeableness. This result is important because it demonstrates the incremental validity of EC beyond personality traits in the prediction of likeability. Previous studies have already shown the incremental validity of ECs in the prediction of social functioning (e.g., van der Zee and Wabeke, 2004), but the PEC, which was used in the current study, allowed us to examine the incremental validity of ECs in more detail, as two different features of EC were taken into account.

Finally, and most importantly, our study yielded new insights into the determinants of likeability, as it demonstrates how extraversion and interpersonal EC combine to influence success in achieving peer acceptance. Specifically, extraversion predicted greater likeability among adolescents with high interpersonal EC but not among adolescents with low interpersonal EC. The opposite was also true: interpersonal EC predicted greater likeability among adolescents with high extraversion but not among adolescents with low extraversion. Why is the combination of high extraversion and high interpersonal EC crucial to increasing peer acceptance?

We believe that extraversion gives adolescents the motivation to create relationships with their peers (Lucas and Diener, 2001; Ciarrochi and Heaven, 2009), while EC provides the skills needed to maintain these relationships (e.g., Lopes et al., 2004, 2005). As a result, it may affect success in attaining peer acceptance. Extroverts are “the life of the party”, outgoing, cheerful and action-oriented individuals, who are likely to respond enthusiastically to exciting challenges (Costa and McCrae, 1992; Lucas and Diener, 2001). These characteristics are definitely an advantage in a classroom setting, as highly extroverted adolescents attract more attention and are positively evaluated by their peers (Ciarrochi and Heaven, 2009). Nevertheless, as our study demonstrated, being an extrovert is not enough. In order to achieve peer acceptance, extroverted adolescents must also have sufficient interpersonal EC at their disposal: they have to be able to identify appropriately, understand, express, regulate and utilize the emotions of others (Brasseur et al., 2013). Alternatively, we may argue that being an emotionally competent adolescent is not enough to be liked. Adolescents who want to gain their peers’ acceptance also need to be active and socially approachable; they simply have to enjoy being with people. Taken together, our results clearly suggest that adolescents need to be both extrovert and possess high interpersonal EC to be judged highly likeable by their peers.

Our study contributes to the constantly accumulating evidence on the effects of an individual’s dispositions on peer-rated likeability in adolescence. First, to the best of our knowledge, none of the previous studies have evaluated the effects of two independent dimensions of EC (i.e., intra- and interpersonalECs) on likeability. Second, the current study is the first to examine the interactive effects of personality traits and EC on likeability. Third, our study provides a possible explanation for the disparity in earlier studies examining the personality–likeability relationship. Fourth, as our research was carried out in Poland, a country undergoing a socio-economic transition from collectivistic to individualistic society (Brycz et al., 2015), it nicely complements previous studies, conducted mainly among North American, Dutch and English adolescents, functioning in individualistic rather than collectivistic societies (Hofstede et al., 2010). It appears that the links between personality and likeability, and between EC and likeability are similar in different countries or at least in different European countries, regardless of their cultural orientations.

There are several limitations to the current study that should be acknowledged. First, the present study used a cross-sectional design; hence, statements about causal relationships should be put forward with caution. Although we implied a certain causal order for the variables (i.e., peer-rated likeability resulted from the adolescents’ personality traits and ECs), other causal directions are possible as well (e.g., likeability as an antecedent of personality traits and ECs). This issue is especially evident when we consider that not only relationships with peers, but also personality traits, including trait EI, are subject to change throughout development (Lamb et al., 2002; Keefer et al., 2013). Future longitudinal studies might clarify the associations demonstrated in the current study. Second, in this study we referred to likeability, which, as alluded to before, represents only one feature of social status in a peer group. Future research is needed to determine the role of ECs in predicting popularity among peers. Third, it should be noted that in comparison to the main effects, the percentage of explained variance attributable to the role of the extraversion × interpersonal EC interaction in predicting peer-rated likeability was rather modest (0.03). Nevertheless, researchers have argued that even a 1% contribution to the total variance should be considered important as the estimation of interactions is generally low (McClelland and Judd, 1993). Fourth, the study participants filled in the questionnaires in the order fixed, which allowed us to separate the anonymous part of the study (personality traits and EC) from its non-anonymous part (sociometric nominations). Although this procedure provided the participants with greater comfort during data collection, it also increased the likelihood of response bias and, for this reason, should be considered a limitation of the study.

Despite the limitations noted above, our results bear several practical implications for the improvement of adolescents’ position and acceptance within their peer group. They suggest that encouraging “rejected” adolescents to reach out to others in an extrovert fashion is necessary but insufficient to increase their acceptance and likeability. Improving their interpersonal EC is also necessary. There is, indeed, accumulating evidence showing that ECs can be lastingly increased via training programs targeting the core ECs (identification, understanding, expression, regulation and use of emotions) (for a review, see Mikolajczak and Peña-Sarrionandia, 2015). Efficient programs to increase intrapersonal EC in adolescents have been successfully developed in recent years (e.g., INTEMO in Spain; Castillo et al., 2013; RULER in the US; Brackett et al., 2012). The current results suggest that it could be particularly beneficial to adolescents if these programs were to extend their focus on interpersonal EC too.

## Author Contributions

DS developed the study design, performed the data collection, and the data analysis. DS and MM contributed to data interpretation and writing the manuscript, and approved the final version of the manuscript for submission.

## Conflict of Interest Statement

The authors declare that the research was conducted in the absence of any commercial or financial relationships that could be construed as a potential conflict of interest.
